# Supplementary material for: Warming induces short-term phenological shifts in pollinator-plant interactions that enhance larval development in honey bee
Source: PLoS One. 2024 Dec 3;19(12):e0314791. doi: 10.1371/journal.pone.0314791 (PMC11614231; doi:10.1371/journal.pone.0314791)
Supplement: S1 File — (DOCX) [file pone.0314791.s001.docx]

**Supporting information**


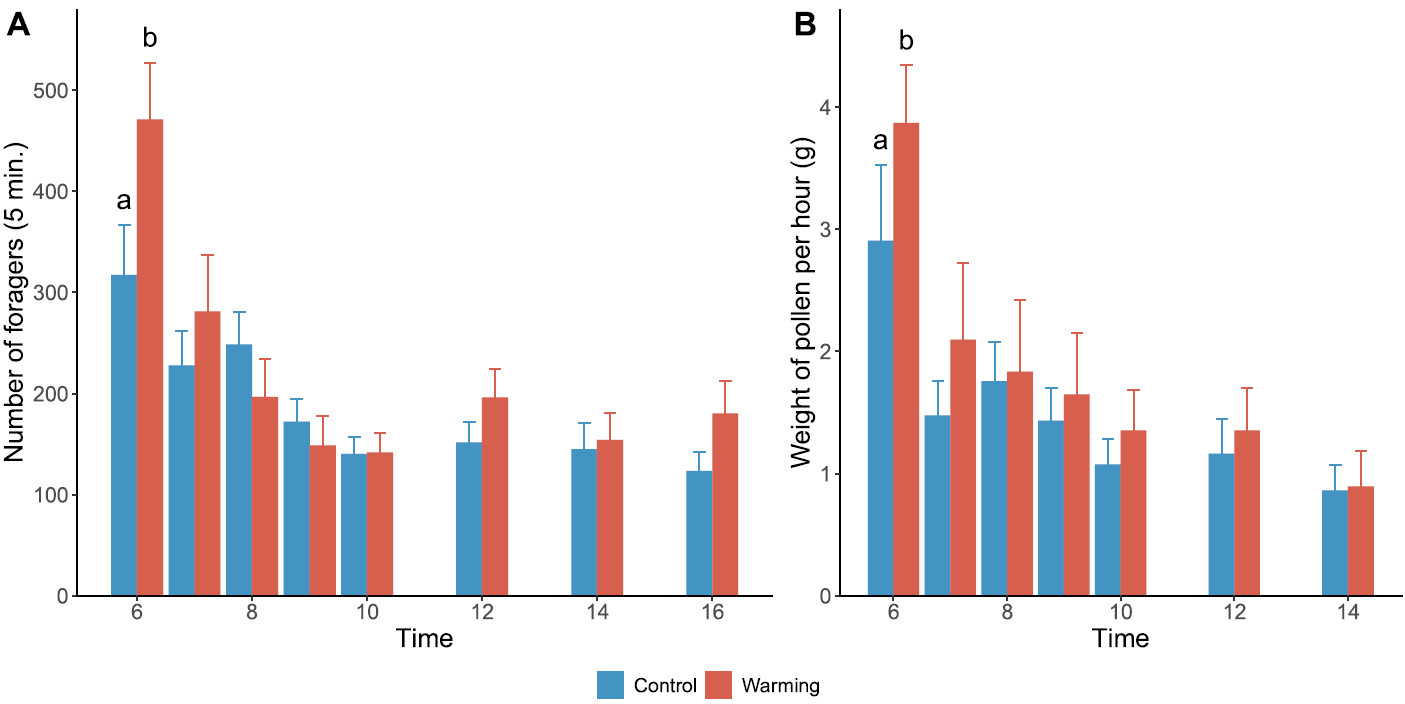


Fig. S1. Temperature treatment impact on bee foraging behavior and pollen load weight. The effect of temperature treatment on (A) the number of foragers departing from beehives and (B) the weight of pollen loads over the time of day. The letters at 6:00 denote significant statistical difference between the control and warming treatments; other times showed no significant difference. Means with standard error are shown.


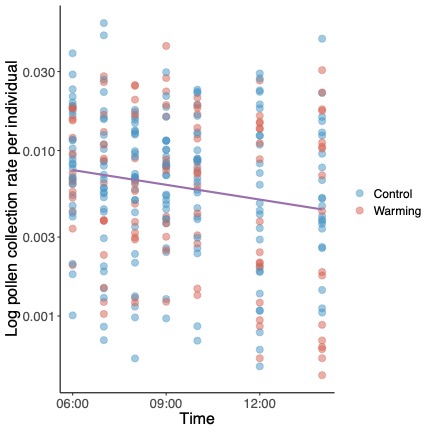


**Fig. S2. The effect of temperature treatment on the pollen collection rate per individual over the time of day.** Lines are predicted relationships from generalized linear mixed models.

**
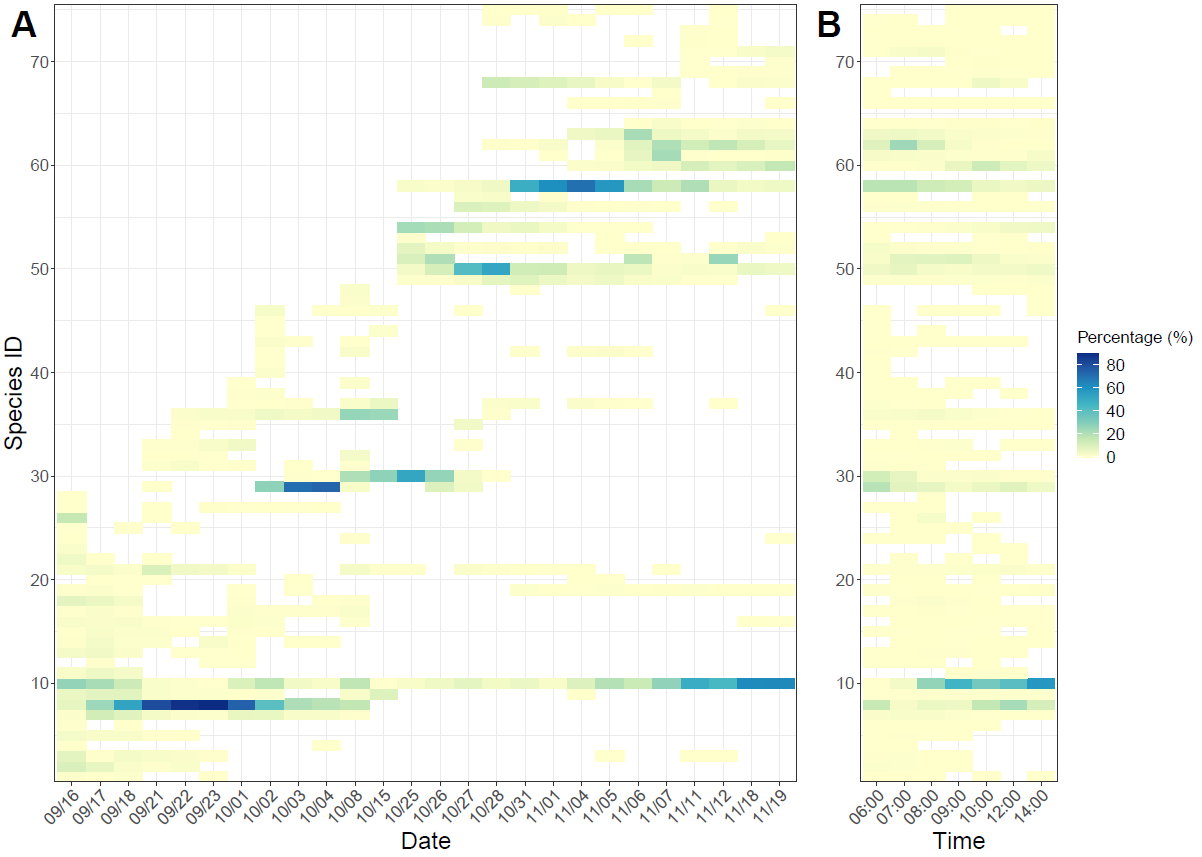
**

Fig. S3. Short-term temporal changes in bee pollen abundance. Temporal patterns of bee pollen abundance of different plant species across (A) days (abundance from 6:00 to 16:00 was summed) and (B) different times of the day. The most abundant species was 28.0% *B. pilosa* (species ID: 10), the second was 12.3% *Koelreuteria elegans* (species ID: 8), followed by 9.8% Bauhinia x blakeana (species ID: 58).


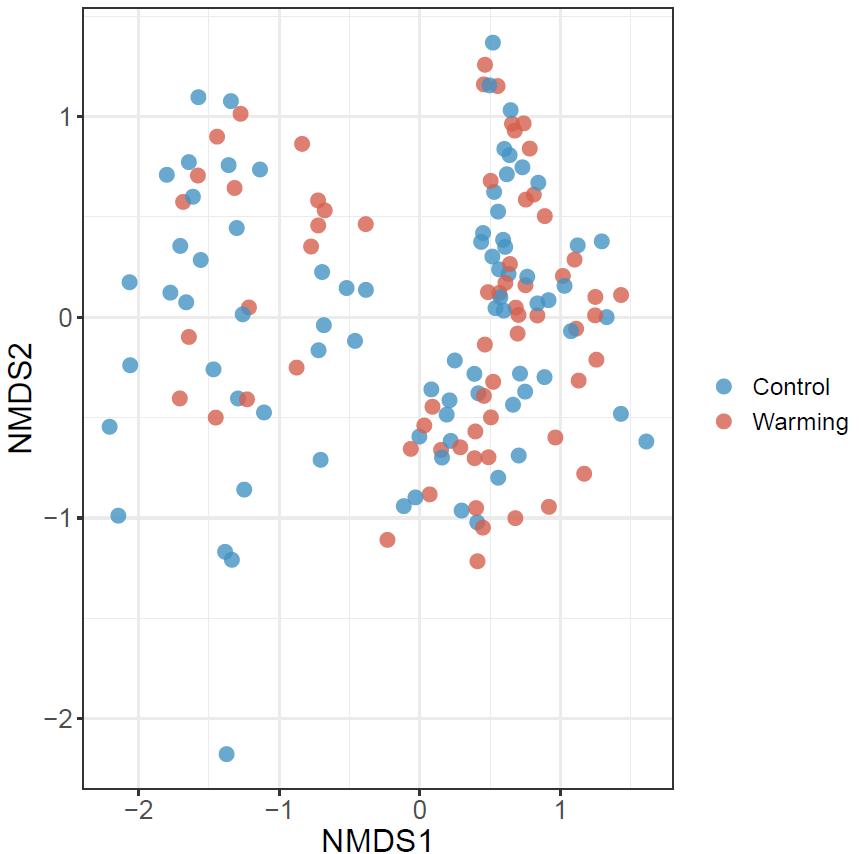


**Fig. S4. Non-metric multidimensional scaling (NMDS) plot of bee pollen composition.** No significant difference was found between the control and warming treatment groups.


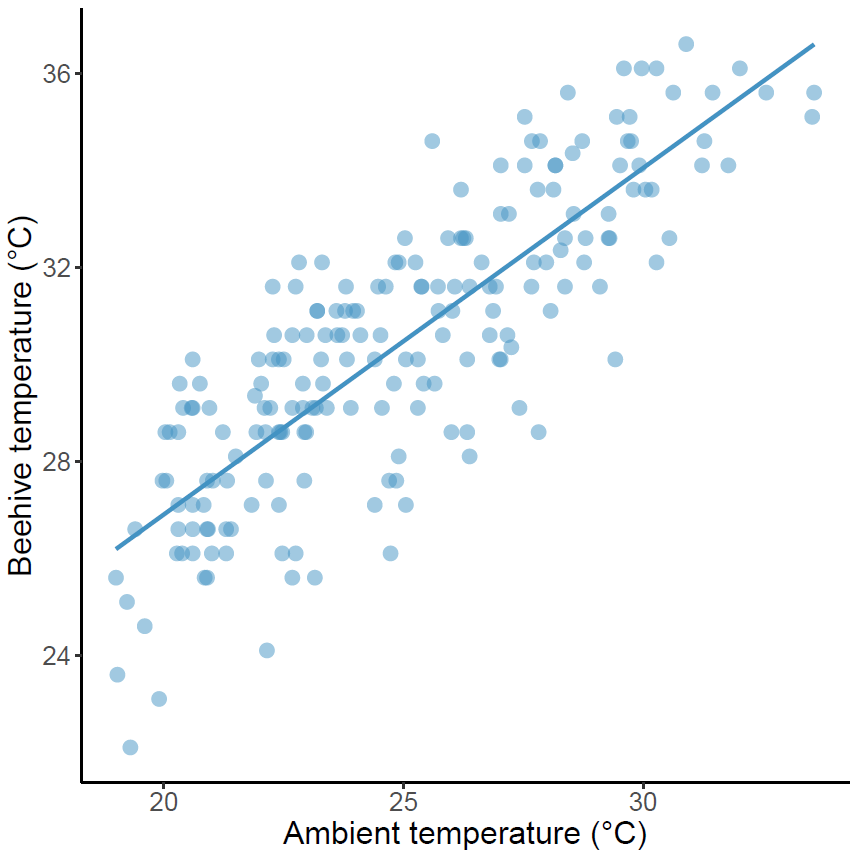


**Fig. S5. The relationship between ambient temperature and beehive temperature.** The line indicates predicted relationship from a generalized linear mixed model.

**
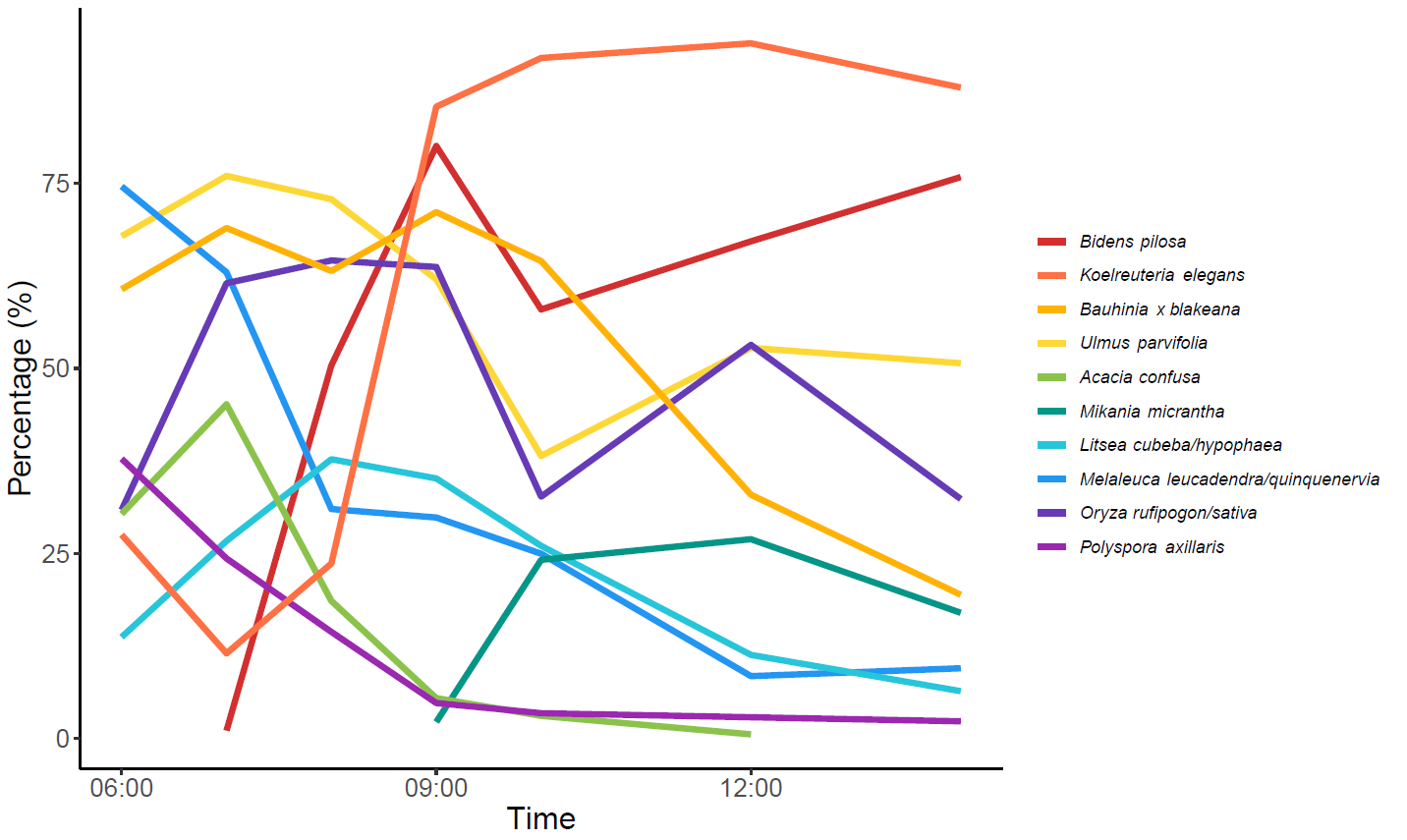
**

**Fig. S6.** **Temporal patterns of pollen diversity.** The percentage of different pollen (top ten most abundant plant species) collected by bees over the time of day over the whole period of field experiment. 6:00 equals to 6:00-7:00, 7:00 equals to 7:00-8:00, etc.

**Fig. S7. An example of experimental set-up to test for the warming effect on bee foraging behavior.** (A) The behaviors were recorded by a camera (Canon IXUS 285 HS) positioned from the top of the landing platform. (B) The heating pad was placed inside the beehive (on top of the brood combs).

| **Table S1. Ten most abundant plant species identified by DNA barcoding and the rest of plant species that were distinguished based on differences in pollen morphology.**   \| **Species ID** \| **Percentage** \| **Identified species** \| \| --- \| --- \| --- \| \| 10 \| 27.968% \| *Bidens pilosa* \| \| 8 \| 12.293% \| *Koelreuteria elegans* \| \| 58 \| 9.752% \| *Bauhinia x blakeana* \| \| 29 \| 8.500% \| *Ulmus parvifolia* \| \| 62 \| 5.996% \| *Acacia confusa* \| \| 60 \| 5.064% \| *Mikania micrantha* \| \| 51 \| 4.042% \| *Litsea cubeba/hypophaea* \| \| 30 \| 3.813% \| *Melaleuca leucadendra/quinquenervia* \| \| 50 \| 3.727% \| *Oryza rufipogon/sativa* \| \| 63 \| 2.191% \| *Polyspora axillaris* \| \| 54 \| 1.848% \| - \| \| 61 \| 1.456% \| - \| \| 68 \| 1.188% \| - \| \| 36 \| 1.172% \| - \| \| 7 \| 0.831% \| - \| \| 52 \| 0.820% \| - \| \| 49 \| 0.784% \| - \| \| 21 \| 0.649% \| - \| \| 71 \| 0.619% \| - \| \| 9 \| 0.462% \| - \| \| 59 \| 0.348% \| - \| \| 56 \| 0.312% \| - \| \| 2 \| 0.311% \| - \| \| 3 \| 0.259% \| - \| \| 64 \| 0.249% \| - \| \| 26 \| 0.239% \| - \| \| 18 \| 0.236% \| - \| \| 19 \| 0.207% \| - \| \| 37 \| 0.202% \| - \| \| 33 \| 0.164% \| - \| \| **Table S1. (continued)** \| \| \| \| **Species ID** \| **Percentage** \| **Identified species** \| \| 16 \| 0.158% \| - \| \| 17 \| 0.151% \| - \| \| 42 \| 0.140% \| - \| \| 55 \| 0.139% \| - \| \| 69 \| 0.137% \| - \| \| 14 \| 0.125% \| - \| \| 11 \| 0.123% \| - \| \| 13 \| 0.117% \| - \| \| 5 \| 0.094% \| - \| \| 31 \| 0.093% \| - \| \| 15 \| 0.082% \| - \| \| 70 \| 0.079% \| - \| \| 22 \| 0.068% \| - \| \| 35 \| 0.066% \| - \| \| 32 \| 0.064% \| - \| \| 74 \| 0.055% \| - \| \| 46 \| 0.054% \| - \| \| 66 \| 0.051% \| - \| \| 43 \| 0.045% \| - \| \| 57 \| 0.040% \| - \| \| 65 \| 0.036% \| - \| \| 53 \| 0.035% \| - \| \| 72 \| 0.033% \| - \| \| 27 \| 0.032% \| - \| \| 24 \| 0.031% \| - \| \| 73 \| 0.029% \| - \| \| 1 \| 0.027% \| - \| \| 38 \| 0.026% \| - \| \| 48 \| 0.025% \| - \| \| 23 \| 0.024% \| - \| \| 39 \| 0.022% \| - \| \| **Table S1. (continued)** \| \| \| \| **Species ID** \| **Percentage** \| **Identified species** \| \| 12 \| 0.018% \| - \| \| 20 \| 0.017% \| - \| \| 47 \| 0.016% \| - \| \| 40 \| 0.015% \| - \| \| 75 \| 0.013% \| - \| \| 6 \| 0.010% \| - \| \| 44 \| 0.009% \| - \| \| 25 \| 0.008% \| - \| \| 67 \| 0.003% \| - \| \| 41 \| 0.003% \| - \| \| 45 \| 0.002% \| - \| \| 28 \| 0.002% \| - \| \| 4 \| 0.002% \| - \| \| 34 \| 0.001% \| - \| \| Others \| 1.978% \| - \| |  |  |  |
| --- | --- | --- | --- | --- | --- | --- | --- | --- | --- | --- | --- | --- | --- | --- | --- | --- | --- | --- | --- | --- | --- | --- | --- | --- | --- | --- | --- | --- | --- | --- | --- | --- | --- | --- | --- | --- | --- | --- | --- | --- | --- | --- | --- | --- | --- | --- | --- | --- | --- | --- | --- | --- | --- | --- | --- | --- | --- | --- | --- | --- | --- | --- | --- | --- | --- | --- | --- | --- | --- | --- | --- | --- | --- | --- | --- | --- | --- | --- | --- | --- | --- | --- | --- | --- | --- | --- | --- | --- | --- | --- | --- | --- | --- | --- | --- | --- | --- | --- | --- | --- | --- | --- | --- | --- | --- | --- | --- | --- | --- | --- | --- | --- | --- | --- | --- | --- | --- | --- | --- | --- | --- | --- | --- | --- | --- | --- | --- | --- | --- | --- | --- | --- | --- | --- | --- | --- | --- | --- | --- | --- | --- | --- | --- | --- | --- | --- | --- | --- | --- | --- | --- | --- | --- | --- | --- | --- | --- | --- | --- | --- | --- | --- | --- | --- | --- | --- | --- | --- | --- | --- | --- | --- | --- | --- | --- | --- | --- | --- | --- | --- | --- | --- | --- | --- | --- | --- | --- | --- | --- | --- | --- | --- | --- | --- | --- | --- | --- | --- | --- | --- | --- | --- | --- | --- | --- | --- | --- | --- | --- | --- | --- | --- | --- | --- | --- | --- | --- | --- | --- | --- | --- | --- | --- | --- | --- | --- | --- | --- | --- | --- | --- | --- | --- | --- | --- | --- | --- | --- | --- | --- | --- | --- | --- | --- | --- | --- |

Others refer to unidentified pollen by morphological traits.

**Table S2. Four different pollen composition treatments used in the larval development experiment.**

| **Species ID** | **Control** | **Warming** | **Warming plus** | ***Bidens* only** |
| --- | --- | --- | --- | --- |
| 10 | 38.00% | 47.00% | 55.00% | 100.00% |
| 60 | 15.33% | 13.10% | 11.12% | 0.00% |
| 62 | 15.18% | 12.98% | 11.02% | 0.00% |
| 51 | 8.57% | 7.33% | 6.22% | 0.00% |
| 58 | 8.52% | 7.29% | 6.19% | 0.00% |
| 50 | 5.22% | 4.46% | 3.79% | 0.00% |
| 63 | 3.24% | 2.77% | 2.35% | 0.00% |
| Others | 1.50% | 1.28% | 1.09% | 0.00% |
| 52 | 1.07% | 0.91% | 0.77% | 0.00% |
| 68 | 1.02% | 0.87% | 0.74% | 0.00% |
| 69 | 0.45% | 0.39% | 0.33% | 0.00% |
| 64 | 0.44% | 0.38% | 0.32% | 0.00% |
| 19 | 0.32% | 0.27% | 0.23% | 0.00% |
| 61 | 0.26% | 0.22% | 0.19% | 0.00% |
| 70 | 0.26% | 0.22% | 0.19% | 0.00% |
| 72 | 0.11% | 0.09% | 0.08% | 0.00% |
| 73 | 0.10% | 0.08% | 0.07% | 0.00% |
| 49 | 0.09% | 0.08% | 0.07% | 0.00% |
| 74 | 0.09% | 0.08% | 0.07% | 0.00% |
| 16 | 0.09% | 0.08% | 0.06% | 0.00% |
| 24 | 0.08% | 0.07% | 0.06% | 0.00% |
| 66 | 0.03% | 0.02% | 0.02% | 0.00% |
| 46 | 0.01% | 0.01% | 0.01% | 0.00% |
| 3 | 0.01% | 0.01% | 0.01% | 0.00% |
| 53 | 0.01% | 0.01% | 0.00% | 0.00% |
| 75 | 0.01% | 0.01% | 0.00% | 0.00% |
| 37 | 0.00% | 0.00% | 0.00% | 0.00% |

Others refer to unidentified pollen by morphological traits.
