# Supplementary material for: Warming induces short-term phenological shifts in pollinator-plant interactions that enhance larval development in honey bee
Source: PLoS One. 2024 Dec 3;19(12):e0314791. doi: 10.1371/journal.pone.0314791 (PMC11614231; doi:10.1371/journal.pone.0314791)
Supplement: Attachment — Submitted filename: PONE-D-24-21139_CommentsToAuthors_20240719.pdf [file pone.0314791.s002.pdf]

<u>Comments to the authors</u>
Manuscript PONE-D-24-21139

Overview:

This paper represents an interesting and novel contribution to our understanding of how climate change-induced warming may influence honeybee behavior and fecundity, as well as flowering timing. The study provides evidence that in the early morning, warming will cause honey bees to forage at higher rates and at higher efficiencies relative to controls, but that warming has no effect on the composition of pollen collected by honeybees. In addition, the study demonstrates that warming advances peak anthesis timing. Finally, it shows that a higher ratio of pollen from a focal species improves larval efficiency. To my knowledge, this is the first paper to address the impact of warming on the time of day of honeybee activity and their primary forage species within the system.

However, I feel that there are several issues with the current version of the manuscript that need to be addressed prior to publication. Principally, the design of the study and language throughout the paper seem to imply that increased temperature will coincide with an increase in prevalence of the pollen of *Bidens pilosa*; however, to my knowledge, this connection was not directly studied. Second, I am concerned that there was only a single beehive used for each treatment. Though I recognize that this was in part ameliorated by the alternation of warming treatments, it remains an issue that is insufficiently addressed in the body of the manuscript. Third, the statistical approaches taken in some areas of the analysis could be improved. Finally, I have several line edits that I have separated by section that may help improve the flow and readability of the manuscript.

I thank the authors for their consideration of these comments. I hope to help assist with this manuscript again in the future.

Title and abstract:

The title and abstract are mostly well written, but I feel that they overstate the results of this paper. Though it is used throughout the title and abstract, I believe that the term ‘phenological shifts’ is only tenuously connected to the findings of this study. The term ‘timing’ may be more appropriate in some cases. In addition, given that composition of pollen collected by bees did not vary significantly between treatments and that both bee and flower timing were advanced by warming, I am unsure as to why the authors state that climate change may affect larval development by changing pollen provisioned to them.

L21: ‘altering their phenologies’- please clarify if both or one taxa’s phenologies are being altered
L22: ‘short-term’- here, it may be helpful to clarify what length of time is being referred to. E.g. ‘within-day’
L23: ‘this issue’- rephrase to be more specific
L26: ‘pollen composition’- this term is used very often throughout the manuscript, and should be clarified in the abstract and main text. Malagnini et al. (2022) do a great job of doing this (https://doi.org/10.3389/fsufs.2022.865368)
L28: ‘studied’- the authors did not directly study these plants, so it may be better to clarify that these were the most commonly represented species in the pollen collected by bees. Also, I suggest adding a smoother transition between these sentences, like “Further”

L29: ‘in the diet’- add ‘pollen’ before this
L30-31: I am unclear on how this study provides evidence for a disruption in interactions, as the timing of both species was advanced
L30: If this sentence structure is maintained, I suggest changing ‘highlights’ to ‘demonstrates’
L31: Suggest changing ‘phenology’ to ‘timing’
L32: Omit ‘the fitness of’

Introduction:

The introduction is mostly well-written and clear. However, the first and second paragraphs of the introduction could be distilled and merged as there are several redundancies among them. Alternatively, they could be made more disparate by emphasizing certain concepts in each paragraph. Further, the fourth paragraph emphasizes phenological shifts, but does not discuss how climate change may influence within-day foraging and flowering timing. The authors may benefit from looking for literature on diel rhythms, 24-hour timing, and circadian clocks of pollinators and flowers to provide more thorough background on the drivers of insect activity and flowering timing. For example, please see the following papers:
Bloch et al. 2017- https://doi.org/10.1098/rstb.2016.0256
Steen et al. 2016- https://doi.org/10.1111/2041-210X.12654
Auffray et al. 2017- https://doi.org/10.1093/jisesa/iex018

Other several important parts of the study are insufficiently supported by the introduction, including the drivers of pollen load size and plant species composition, as well as the influence of diet on larval development efficiency. These concepts need to be addressed in the introduction. Please find my line edits below.

L39: Double space before ‘(Traill’
L42-43: This is phrased to imply that general disruptions to plants and pollinators cause phenological mismatches- suggest rephrasing to ‘...interactions by causing phenological mismatches.’
L46-47: I suggest rephrasing to ‘A reduction in effective pollination could reduce seed set in plants that rely on pollinator outcrossing for reproduction, thereby reducing the total quantity of pollen and nectar rewards available to pollinators in future seasons’, then cite this concept.
L50: ‘effectively increase’ to ‘facilitate’
L50-51 and L55-62: These sentences are more related to the sentences on L42-45 than those on L51-52. If L50-51 and L55-62 are merged into the first introduction paragraph, flow would improve. Alternatively, the authors could introduce the idea that climate change causes phenological mismatches and detriments pollination in paragraph 1, then emphasize its effect on honeybees in paragraph 2.
L58: I suggest adding ‘or be reduced’ after ‘coincide’
L64: I suggest adding ‘most’ after ‘To date,’
L65: Please clarify the meaning of ‘spring activities’
L67: ‘fluctuations’- please describe what fluctuations these refer to. Also, I’m not sure that it is entirely true that these responses remain unclear- the next sentence appears to address some of these responses. It may improve clarity to rephrase to say that these responses are understudied.
L70-70: I suggest changing the part of this sentence after ‘et al., 2020)’ to ‘causing bees to seek water for use in evaporative cooling’
L71: ‘The foraging activity’ of what species?
L72: Add ‘within-day’ before ‘variations

L74-75: This same concept was stated on L69-70
L75-76: Repetitive with previous sentences
L76-80: This idea would be more useful if it were tied it into the hypothesis on L104-106
L78: Please cite (Reddy et al. 2015) here as well
L80-82: This feels contradictory to the phrase on L66-67
L82-83: I suggest developing this idea more to provide more support for the anthesis onset component of the paper
L86: This may be a good place to describe the meaning of pollen composition (see my comment about L26)
L87: This is setting the reader up for a paper on phenology, not on daily fluctuations in resource availability
L92: This raises the question of whether honeybees eat pollen or if they only use it to provision their larvae- this would be an interesting tidbit to add
L93: I suggest changing 'study experimentally manipulating' to 'a study that experimentally manipulated'
L101-103: These questions may better prepare the reader to understand this paper if they are edited to align directly with the individual components of the experiment. In addition, I suggest rephrasing these to questions- e.g., '(1) does warming affect bee foraging behaviors
L104: Meaning of 'through' unclear
L104-106: Add 'would' after 'warming' and describe what aspect of foraging behavior would increase/decrease
L109: 'of' to 'in' and 'predict' to 'expected to find'

Results:

The results are well organized. However, I am confused by the interpretation of some model results, especially those on L117-127. I do not understand why the square and cube of time were included in the model. This reasoning should be included somewhere in the text. Please find my line edits below.

L113 and L144: Suggest changing 'Warming effect' to 'Effect of warming'
L117: Because time did not significantly interact with treatment, I do not feel that this argument can be made as strongly as it is.
L118: Suggest rephrasing to 'that at 6:00, warming induced…'
L119: 'departing' to 'to depart'
L122: 'Note that the' to 'These'
L124: 'Specifically' to 'Again'
L127: 'regardless of the' to 'and was not significantly different between'
L132: Change to 'The species with the ten highest relative…'
L134-135: replace 'highest' with 'most abundant'
L135: Reference not found
L136: Please describe the meaning of 'periodically'- how often did most species appear? On what percentage of days?
L137: Was *B. pilosa* pollen collected by all bees every day? Please clarify.
L138-139: Suggest rephrasing to include more detail- e.g., 'The composition of pollen varied by time of day. Between 6:00 and 7:00, pollen of *B. pilosa* represented an average of __% of pollen loads collected by bees, but after 8:00, *B. pilosa* pollen represented an average of only __% of pollen loads' or similar
L147: Please describe the meaning of 'anthesis onset peak' and how this was calculated
L147-148: Please include a p-value and other relevant statistics to support this claim

L148: Please describe the meaning of or otherwise clarify ‘higher number of anthesis onset’
L147-149: Was this trend significant?
L151-155: The reasoning behind including this section should be clarified somewhere in the text, because as stated on lines 140-142, temperature treatment did not significantly affect the composition of the pollen collected by bees.
L153: This sentence could be made more clear by stating that increased *B. pilosa* pollen increased larval efficiency. It seems to me that there may be a threshold percentage of *B. pilosa* pollen that improves larval efficiency (between its concentrations in the control and warming treatments) that may be of interest.

Discussion:

The discussion addresses most of the components of the paper, but in my view, does not thoroughly analyze many of the important findings. In particular, the authors do not discuss their findings that the composition of pollen collected by bees did not differ between treatments. Further, I feel that some unsubstantiated claims are made that should be removed. Namely, the authors state that they have evidence that warming can lead to shifts in the composition of pollen collected by pollinators, which, by my interpretation, is in contradiction with their data.

L160: The term ‘Consequently’ does not seem to fit here
L162-164: This reasoning does not make sense to me, and seems unsubstantiated by the data. This study showed that there was no significant difference in the composition of pollen collected by treatment, and, further, both bees and flowers became active earlier under warming. Thus, I don’t understand why the authors claim that a shift in flowering will change the pollen available to bees under warming. I may be misunderstanding but this should be clarified.
L170-171: Suggest rephrasing to ‘Environmental factors that are known to affect bee foraging – such as temperature and solar radiation – vary…’ Then, it would improve flow to connect this sentence with the rest of the paragraph by, for example, emphasizing the role of temperature in shaping diel rhythms in bee behavior.
L173: Suggest omitting ‘clearly’
L177: Please clarify the meaning of ‘the effect of time’- on what, and at what scale?
L178-179: I suggest explaining how temperature may not affect bee activity. Otherwise this statement seems to contradict many of the findings in this study.
L179-181: I suggest rephrasing to ‘A more comprehensive understanding of the relative importance of within-hive and ambient temperature to bee foraging behavior could be developed by…’ then describe what kind of study could be done to this end.
L182-183: I feel that this sentence would fit better earlier in the paragraph.
L183-184: I suggest replacing ‘early in the morning’ with ‘at 6:00’ and remove ‘at 6:00’ from later in the sentence
L185: If true, I suggest replacing ‘not evident’ with ‘not captured by the range of temperatures in this study’
L185-186: I suggest omitting ‘Turning to foraging efficiency per individual’, the replace ‘the efficiency’ with ‘foraging efficiency’
L190: I suggest adding ‘early-foraging’ after ‘since
L191: I suggest add ‘pollen’ before ‘quantities’
L194-197: I suggest rephrasing this sentence to improve clarity, perhaps by moving the information on the generality of honey bees to a separate sentence.
L197-198: Because this was not tested in this study, it may be helpful to include a reference here.
L199: For clarity, please add ‘floral’ before ‘resources’ and ‘landscape’

L200-202: I am unclear on how the authors have come to this conclusion based on the results of this study.
L208-210: I suggest citing these concepts.
L216-219: I suggest connecting this sentence to the remainder of the paragraph
L216: Please change ‘nutritional values’ to ‘nutrients’
L220-222: I suggest rephrasing to ‘development of honey bees [if true] due to the lack of necessary nutrients (Filipiak et al., 2017), although access to a single flowering species with protein-rich pollen may be more important than high floral diversity for larval growth…’
L227-228: This seems important, and it may be helpful to address this concept sooner.
L229-231: Please rephrase to emphasize that this study’s findings are in contrast with those of Tasei and Aupinel.
L233: I suggest clarifying what is meant by ‘changes in’
L234: I suggest replacing ‘are determined by several factors, such as’ with ‘change across’
L235-236: I suggest rephrasing to ‘Since __ months passed between our field and larvae feeding experiments…’
L237: I suggest omitting ‘in’, add ‘the’ before ‘larval’, and change ‘experiments’ to ‘experiment’
L238: I suggest adding ‘that were more common’ after ‘that’
L239: I suggest omitting this line as it is implied
L241: Please clarify the meaning of ‘seasonally consistent’
L241-242: Please describe how this may occur
L243-244: Please describe what is meant by ‘larvae grew heavier’
L244: Expand on how a pollen-only diet could affect larval resilience
L245: Please describe what ‘other developmental factors’ should be evaluated
L246: There seems to be text missing after ‘consequences’
L249: This section does a good job in addressing the small sample size of this study, but does not touch on the issue of any lingering effects in hives the day after they were used in the opposite treatment. To me, this seems like an important angle.
L254: To me, ‘physiological changes’ include an increase in metabolism driven by high temperatures. Perhaps the authors intended to discuss acclimatization to warming.
L258: I suggest rephrasing to ‘hives to test the robustness of our findings’
L261: I suggest clarifying what has occurred over the past decade that has made studying these trends more important
L265-267: From my reading of this manuscript, I disagree that the data, as currently presented, supports the idea that warming changes the composition of pollen collected by honey bees.

Methods:

The methods are mostly clear. However, I have several line edits and concerns about pseudoreplication and model selection, which I describe below.

L273: Though this was addressed in the discussion, I am still concerned that there is a high level of pseudoreplication in this study given that only two hives were used
L275: Please state which pollen substitutes were used
L277: Please describe why hives were heated for 20 hours and not 24
L277: ‘beehive’ should be plural here, I believe
L292: Why one to two hours, and not every hour? Please clarify.
L293: Please add the number of total days of the experiment here for clarity
L294-296: Please describe how observers knew which bees were starting orientation flights
L298: I suggest omitting the ‘the’ before ‘ImageJ’

L299: To cite ImageJ, I believe one should use Schneider et al. 2012 (https://doi.org/10.1038/nmeth.2089). Also, I suggest starting a new paragraph at the end of this sentence.
L300-301: I suggest describing what pollen traps are and how much pollen they allow one to observe of what the bees have collected
L302: Please explain why pollen was collected every one to two hours at these different intervals
L304: Please clarify the meaning of ‘standardized per hour’
L305: Please describe what an observation entails in this context
L307: I suggest replacing ‘refrigerator’ with ‘freezer’ and clarifying what further investigation was performed
L317: ‘pollen’- does this refer to a pollen grain or the entirety of that species’ pollen? Please clarify.
L320: Reference not found
L340: I suggest omitting ‘the’
L344: Please include the family that *B. pilosa* belongs to
L346: Reference not found
L349: Please describe why these growing conditions were chosen
L351: Please clarify whether plants were moved on the 17th day of seedling growth or 17 days after planting
L355-356: Please describe how these temperatures were chosen
L357: Suggest changing ‘would be’ to ‘were’
L358-360: Please state how much fertilizer was used
L360: Please clarify how many individuals had begun flowering when the observations began
L362: I suggest rephrasing to ‘that had just started their first bloom or finished their last bloom’
L363: Why was there a difference in the number of individuals in control versus warming?
L366: ‘anther’ should be plural here
L375: Please clarify what ratio the semi-artificial diet was in
L378: Reference not found
L381: Please clarify how this equation was developed, and why it is of use here
L395: Please clarify what further experiments that are referenced. If they are not otherwise mentioned in this study then I would suggest removing this.
L397: Here and elsewhere I believe this should be formatted as ‘from Hendriksma et al. (2011).’
L406: ‘could’ to ‘was used to’ if this is the case
L407: Please describe why there was a change in feeding regimen after day 1
L411: Extra space after ‘-20’, and ‘refrigerator’ should be ‘freezer’
L413: ‘According’ is not the appropriate term here from my understanding
L413: Please clarify what diet was given
L414: Please describe why the volume of food was increased over time
L416: Please describe the intervals at which the larvae were fed
L417: Omit ‘meticulously’
L419: I do not think that parentheses are needed here
L425: This citation is out of date, I believe
L429: Why was a VIF of <5 chosen? This seems high. Please cite.
L424-430: Was any model selection performed? Given the large number of predictors, I feel this may be an important step in analyzing these data in a non-biased fashion.
L439-441: This sentence brings up many questions- why was the third degree included, and how did the authors make the decision to reduce this degree? Again, was model selection being used?
L442: Were daily averages of ambient temperature and colony size used? Please clarify.
L443: This analysis needs to be fully described.

L448: Suggest rephrasing to ‘To compare the composition of pollen collected by bees under control…’
L455-461: The authors may also consider testing whether anthesis alone was significantly earlier in warming treatments by simply testing for a difference in average anthesis time between treatments.
L458-459: Please further describe why the second degree was used.
L459: I suggest omitting ‘different’
L464: I suggest omitting ‘the’ before ‘larval’
L466: I suggest omitting ‘Different’

Figures and tables:

Across all figures and tables, please ensure that species names are italicized.

Fig. 1: Standard error shading would help with the interpretation of these curves, as would a more thorough description of how the curves were produced.

Fig. 2: Missing a legend for colors. Please also describe how these curves were fit and add standard error shading.

Fig. 3: Please describe how these curves were fit and add standard error shading. Also, the phrase ‘total number of anthesis onset’ is unclear, please rephrase. Finally, it may be better to include the actual time of peak flowering anthesis.

Fig. 4: For (A), box plots would be more appropriate in my opinion. In the caption, it would be more clear to place the letters (A) and (B) before their respective descriptions. Also, please include a legend describing the colors in (B). Finally, please minimize the overlap among colors between (A) and (B).
