# Supplementary material for: Warming induces short-term phenological shifts in pollinator-plant interactions that enhance larval development in honey bee
Source: PLoS One. 2024 Dec 3;19(12):e0314791. doi: 10.1371/journal.pone.0314791 (PMC11614231; doi:10.1371/journal.pone.0314791)
Supplement: Attachment — Submitted filename: response to reviewer comments.docx [file pone.0314791.s003.docx]

5. Review Comments to the Author

Please use the space provided to explain your answers to the questions above. You may also include additional comments for the author, including concerns about dual publication, research ethics, or publication ethics. (Please upload your review as an attachment if it exceeds 20,000 characters)

Reviewer #1: This paper represents an interesting and novel contribution to our understanding of how climate change-induced warming may influence honeybee behavior and fecundity, as well as flowering timing. The study provides evidence that in the early morning, warming will cause honey bees to forage at higher rates and at higher efficiencies relative to controls, but that warming has no effect on the composition of pollen collected by honeybees. In addition, the study demonstrates that warming advances peak anthesis timing. Finally, it shows that a higher ratio of pollen from a focal species improves larval efficiency. To my knowledge, this is the first paper to address the impact of warming on the time of day of honeybee activity and their primary forage species within the system.

However, I feel that there are several issues with the current version of the manuscript that need to be addressed prior to publication. Principally, the design of the study and language throughout the paper seem to imply that increased temperature will coincide with an increase in prevalence of the pollen of Bidens pilosa; however, to my knowledge, this connection was not directly studied. Second, I am concerned that there was only a single beehive used for each treatment. Though I recognize that this was in part ameliorated by the alternation of warming treatments, it remains an issue that is insufficiently addressed in the body of the manuscript. Third, the statistical approaches taken in some areas of the analysis could be improved. Finally, I have several line edits that I have separated by section that may help improve the flow and readability of the manuscript.

I thank the authors for their consideration of these comments. I hope to help assist with this manuscript again in the future.

Please review my comments in the attached document.

**We are very grateful for reviewer 1 for the very constructive comments. We have accepted most of them with suggested changes. We have also elaborated in details our study design and discussed potential issue of pseudoreplication. We have now responded each specific comment below.**

**Title and abstract:**

Reviewer comment 1:

The title and abstract are mostly well written, but I feel that they overstate the results of this paper. Though it is used throughout the title and abstract, I believe that the term ‘phenological shifts’ is only tenuously connected to the findings of this study. The term ‘timing’ may be more appropriate in some cases. In addition, given that composition of pollen collected by bees did not vary significantly between treatments and that both bee and flower timing were advanced by warming, I am unsure as to why the authors state that climate change may affect larval development by changing pollen provisioned to them.

**Author response 1: Thank you for your insightful comment. We agree that ‘phenological shifts’ traditionally refers to longer-term, seasonal changes. However, our study addresses shifts in the short-term synchrony of biological timing events (e.g., timing of bee foraging and flower anthesis), and thus, we used ‘phenological shifts’ to capture the interconnected timing changes in bees and flowers under warming conditions. We have further clarified our terminology in the revised manuscript, particularly where ‘timing’ is more appropriate.**

**We also appreciate your concern regarding the interpretation of pollen composition and larval development. Since we found no significant difference in pollen composition between control and warming treatments, we understand that discussing pollen-driven impacts on larval development may have been unclear. In our study, we found the advanced effect of warming on the timing of bee foraging (Experiment 1) and flowering (Experiment 2), with the expectation that earlier flowering induced by warming would lead to higher availability of *B. pilosa* pollen for bees during earlier foraging hours. Subsequently, we manipulated pollen compositions that reflect a projected future scenario (Experiment 3) to assess their impacts on larval development. Unfortunately, logistical constraints prevented simultaneous warming of both hives and plants, which we acknowledge as a limitation of our study.**

Reviewer comment 2:

L21: ‘altering their phenologies’- please clarify if both or one taxa’s phenologies are being altered

**Author response 2: By this we meant both the phenologies of plants and pollinators can be altered. We have now made this clear by adding ‘of both parties’ after ‘altering their phenologies’.**

Reviewer comment 3:
L22: ‘short-term’- here, it may be helpful to clarify what length of time is being referred to. E.g. ‘within-day’

**Author response 3: Thank you for your comment. We have rephrased this sentence by adding long-term, seasonal phenological changes, as a contrast to short-term phenological changes. Yet we prefer to use short-term here, but not specify ‘within-day’, since we would like the abstract to begin as general as possible. In addition, we have specified our scale as ‘within-day’ in the following sentences.**

Reviewer comment 4:

L23: ‘this issue’- rephrase to be more specific

**Author response 4: We have now combined this sentence and the next to make our case specific.**

Reviewer comment 5:
L26: ‘pollen composition’- this term is used very often throughout the manuscript, and should be clarified in the abstract and main text. Malagnini et al. (2022) do a great job of doing this (<https://doi.org/10.3389/fsufs.2022.865368>)

**Author response 5: We agree that it needs to be clarified upfront. We have made it clearer by adding ‘, in terms of the relative abundance of pollen from different plant species’.**

Reviewer comment 6:
L28: ‘studied’- the authors did not directly study these plants, so it may be better to clarify that these were the most commonly represented species in the pollen collected by bees. Also, I suggest adding a smoother transition between these sentences, like “Further”

**Author response 6: We have now rephrased this sentence.**

Reviewer comment 7:

L29: ‘in the diet’- add ‘pollen’ before this

**Author response 7: We have now added ‘pollen’.**

Reviewer comment 8:

L30-31: I am unclear on how this study provides evidence for a disruption in interactions, as the timing of both species was advanced

**Author response 8: We agree with you that in our case, disruption is not appropriate. Yet since we have only tested specifically the interactions between bees and *B. pilosa,* what we found here might not be applicable to bee’s interactions with other plant species. Thus, we have rephrased this sentence accordingly.**

Reviewer comment 9:
L30: If this sentence structure is maintained, I suggest changing ‘highlights’ to ‘demonstrates’

**Author response 9: We have changed ‘highlights’ to ‘demonstrates’, which is more appropriate.**

Reviewer comment 10:

L31: Suggest changing ‘phenology’ to ‘timing’

**Author response 10: We thank the reviewer for this comment and prefer to retain the term ‘phenology’ as phenology specifically refers to the timing of biological events, such as flowering and pollinating, in response to environmental changes.**

Reviewer comment 11:
L32: Omit ‘the fitness of’

**Author response 11: We have removed ‘the fitness of’.**

**Introduction:**

Reviewer comment 12:

The introduction is mostly well-written and clear. However, the first and second paragraphs of the introduction could be distilled and merged as there are several redundancies among them. Alternatively, they could be made more disparate by emphasizing certain concepts in each paragraph. Further, the fourth paragraph emphasizes phenological shifts, but does not discuss how climate change may influence within-day foraging and flowering timing. The authors may benefit from looking for literature on diel rhythms, 24-hour timing, and circadian clocks of pollinators and flowers to provide more thorough background on the drivers of insect activity and flowering timing. For example, please see the following papers:

Bloch et al. 2017- https://doi.org/10.1098/rstb.2016.0256 Steen et al. 2016- https://doi.org/10.1111/2041-210X.12654 Auffray et al. 2017- https://doi.org/10.1093/jisesa/iex018

Other several important parts of the study are insufficiently supported by the introduction, including the drivers of pollen load size and plant species composition, as well as the influence of diet on larval development efficiency. These concepts need to be addressed in the introduction. Please find my line edits below.

**Author response 12: Thank you for your valuable feedback! We have carefully followed your suggestions here and in your additional comments to streamline the structure by merging paragraphs and minimizing redundancies. We have expanded the third paragraph in the introduction to illustrate the links between phenological shifts and the potential impact of climate change on plant-pollinator interactions. Additionally, we have strengthened the introduction by providing more background on the factors influencing the number of foragers, pollen load weight, and pollen composition, as well as their implications for larval development. This added context supports our focus on these critical aspects of the study.**

Reviewer comment 13:

L39: Double space before ‘(Traill’

**Author response 13: We have made it a single space.**

Reviewer comment 14:
L42-43: This is phrased to imply that general disruptions to plants and pollinators cause phenological mismatches- suggest rephrasing to ‘...interactions by causing phenological mismatches.’

**Author response 14: We have rephrased it as you suggested.**

Reviewer comment 15:
L46-47: I suggest rephrasing to ‘A reduction in effective pollination could reduce seed set in plants that rely on pollinator outcrossing for reproduction, thereby reducing the total quantity of pollen and nectar rewards available to pollinators in future seasons’, then cite this concept. L50: ‘effectively increase’ to ‘facilitate’

**Author response 15: We appreciate your suggested changes and make the subsequent changes, which flows better now; in previous L50, this sentence does not exist anymore and so this comment is not relevant.**

Reviewer comment 16:

L50-51 and L55-62: These sentences are more related to the sentences on L42-45 than those on L51-52. If L50-51 and L55-62 are merged into the first introduction paragraph, flow would improve. Alternatively, the authors could introduce the idea that climate change causes phenological mismatches and detriments pollination in paragraph 1, then emphasize its effect on honeybees in paragraph 2.

**Author response 16: Thank you for your suggestion. We have considered this modification by integrating L55-62 into paragraph 1. We have also removed previous L50-51 since we have already presented similar concept in paragraph 1 to avoid repetition.**

Reviewer comment 17:

L58: I suggest adding ‘or be reduced’ after ‘coincide’

**Author response 17: We have made the suggested change.**

Reviewer comment 18:

L64: I suggest adding ‘most’ after ‘To date,’

**Author response 18: We have added ‘most’ after ‘To date’.**

Reviewer comment 19:

L65: Please clarify the meaning of ‘spring activities’

**Author response 19: We have replaced the previous reference on bumble bees with an updated ones of honey bees, and have made this term clear.**

Reviewer comment 20:
L67: ‘fluctuations’- please describe what fluctuations these refer to. Also, I’m not sure that it is entirely true that these responses remain unclear- the next sentence appears to address some of these responses. It may improve clarity to rephrase to say that these responses are understudied.

**Author response 20: We agree with you that ‘fluctuations’ is unclear. We have now made it clear as ‘within-day phenological match-mismatch’. We have also replaced unclear with understudied.**

Reviewer comment 21:
L70-70: I suggest changing the part of this sentence after ‘et al., 2020)’ to ‘causing bees to seek water for use in evaporative cooling’

**Author response 21: We have made the suggested change to this sentence.**

Reviewer comment 22:
L71: ‘The foraging activity’ of what species?

**Author response 22: We have added *A. mellifera*.**

Reviewer comment 23:
L72: Add ‘within-day’ before ‘variations

**Author response 23: We have added ‘within-day’.**

Reviewer comment 24:

L74-75: This same concept was stated on L69-70

**Author response 24: We have removed the sentence (previous L74-75).**

Reviewer comment 25:
L75-76: Repetitive with previous sentences

**Author response 25: We have removed this sentence.**

Reviewer comment 26:
L76-80: This idea would be more useful if it were tied it into the hypothesis on L104-106

**Author response 26: We have now referred this work done by Reddy et al. in our hypothesis.**

Reviewer comment 27:
L78: Please cite (Reddy et al. 2015) here as well

**Author response 27: We have now cited it.**

Reviewer comment 28:
L80-82: This feels contradictory to the phrase on L66-67

**Author response 28: We have rephrased L80-82.**

Reviewer comment 29:
L82-83: I suggest developing this idea more to provide more support for the anthesis onset component of the paper

**Author response 29: We noticed that our use of anthesis onset is inappropriate for our focal species *B. pilosa*, which can be misleading; thus, we have replaced this term with flowering.**

Reviewer comment 30:
L86: This may be a good place to describe the meaning of pollen composition (see my comment about L26)

**Author response 30: We have defined our use of pollen composition as the variety of bee pollen collected by bees from different plant species.**

Reviewer comment 31:
L87: This is setting the reader up for a paper on phenology, not on daily fluctuations in resource availability

**Author response 31: Our purpose of setting up this paragraph, focusing on plant phenology, is to act as a contrast of previous paragraphs, which focus on temperature change effect on pollinator activities. Hence, we do not intend to narrow this idea down here to mean daily fluctuations but plant phenology in general.**

Reviewer comment 32:
L92: This raises the question of whether honeybees eat pollen or if they only use it to provision their larvae- this would be an interesting tidbit to add

**Author response 32: Thank you for raising this point. Although we mentioned the role of pollen on bee physiology and larval development here, only larvae feed on the pollen (not adults). By bee physiology we meant that earlier diet of pollen can determine bee physiology as they become adults. We have now clarified this.**

Reviewer comment 33:
L93: I suggest changing ‘study experimentally manipulating’ to ‘a study that experimentally manipulated’

**Author response 33: Thank you – we have made the suggested change.**

Reviewer comment 34:
L101-103: These questions may better prepare the reader to understand this paper if they are edited to align directly with the individual components of the experiment. In addition, I suggest rephrasing these to questions- e.g., ‘(1) does warming affect bee foraging behaviors

**Author response 34: Thank you for the comments. We have rephrased these into three questions. Also, we have referred these three questions alongside the respective experiment 1-3.**

Reviewer comment 35:
L104: Meaning of ‘through’ unclear

**Author response 35: We have replaced ‘through’ with ‘by altering’.**

Reviewer comment 36:
L104-106: Add ‘would’ after ‘warming’ and describe what aspect of foraging behavior would increase/decrease

**Author response 36: We have added ‘would’ after ‘warming’. We have also made clear in the predictions that our use of foraging behavior here refers to the number of foragers and the weight of pollen loads carried by bees.**

Reviewer comment 37:
L109: ‘of’ to ‘in’ and ‘predict’ to ‘expected to find’

**Author response 37: We have made these two changes.**

**Results:**

Reviewer comment 38:
The results are well organized. However, I am confused by the interpretation of some model results, especially those on L117-127. I do not understand why the square and cube of time were included in the model. This reasoning should be included somewhere in the text. Please find my line edits below.

**Author response 38: Thank you for your comment. We included the square and cube of time in the model to capture potential non-linear relationships in the responses of bees and plants over time, which we observed in our data. Including these terms allowed us to model complex, non-linear trends more accurately. The models were evaluated and compared based on their AIC values, and we reported the best-fitting models in the results section. We have clarified this rationale in the methods section to make our approach more transparent.**

Reviewer comment 39:

L113 and L144: Suggest changing ‘Warming effect’ to ‘Effect of warming’

**Author response 39: We have made the suggested changes.**

Reviewer comment 40:
L117: Because time did not significantly interact with treatment, I do not feel that this argument can be made as strongly as it is.

**Author response 40: We felt like there is a misunderstanding here. The statistical analysis suggested a significant interaction between time^3 and warming (*p* = 0.046).**

Reviewer comment 41:
L118: Suggest rephrasing to ‘that at 6:00, warming induced...’

**Author response 41: We have made the suggested changes.**

Reviewer comment 42-47:
L119: ‘departing’ to ‘to depart’
L122: ‘Note that the’ to ‘These’
L124: ‘Specifically’ to ‘Again’
L127: ‘regardless of the’ to ‘and was not significantly different between’
L132: Change to ‘The species with the ten highest relative...’
L134-135: replace ‘highest’ with ‘most abundant’

**Author response 42-47: We have made the suggested changes from these comments.**

Reviewer comment 48:

L135: Reference not found

**Author response 48: This should be Table S1. We have corrected it now.**

Reviewer comment 49:
L136: Please describe the meaning of ‘periodically’- how often did most species appear? On what percentage of days?

**Author response 49: We have provided this information as demonstrated in Fig. S3A. And as the reviewer pointed out, we noticed that our previous description was inaccurate and have thus rephrased this sentence.**

Reviewer comment 50:
L137: Was *B. pilosa* pollen collected by all bees every day? Please clarify.

**Author response 50: We were unable to tell if all bees collected *B. Pilosa* pollen every day since our observation was based on the colony level at different timing.**

Reviewer comment 51:
L138-139: Suggest rephrasing to include more detail- e.g., ‘The composition of pollen varied by time of day. Between 6:00 and 7:00, pollen of *B. pilosa* represented an average of __% of pollen loads collected by bees, but after 8:00, *B. pilosa* pollen represented an average of only __% of pollen loads’ or similar

**Author response 51: Thank you for this suggestion. We have rephrased this sentence as you suggested and have now included the percentage to present the major difference.**

Reviewer comment 52:

L147: Please describe the meaning of ‘anthesis onset peak’ and how this was calculated

**Author response 52: We apologise for not introducing this term clearly. We have rephrased this sentence. Importantly, our use of anthesis onset is not entirely accurate for our focal species *B. pilosa*. We have now made a fundamental change of this term throughout the manuscript.**

Reviewer comment 53:

L147-148: Please include a p-value and other relevant statistics to support this claim

**Author response 53: This claim was made exactly from our significant interaction term between time^2 and temperature treatment (Table 1), which already indicates the shape of the curves, including their peaks, differ between the treatments.**

Reviewer comment 54:

L148: Please describe the meaning of or otherwise clarify ‘higher number of anthesis onset’

**Author response 54: We have changed our use of anthesis onset now.**

Reviewer comment 55:

L147-149: Was this trend significant?

**Author response 55: Yes, the temperature treatment was statistically significant (χ2 28.84, df = 1, p < 0.001), which we have already referred in Table 1.**

Reviewer comment 56:
L151-155: The reasoning behind including this section should be clarified somewhere in the text, because as stated on lines 140-142, temperature treatment did not significantly affect the composition of the pollen collected by bees.

**Author response 56: See also Author response 1.**

Reviewer comment 57:

L153: This sentence could be made more clear by stating that increased *B. pilosa* pollen increased larval efficiency. It seems to me that there may be a threshold percentage of *B. pilosa* pollen that improves larval efficiency (between its concentrations in the control and warming treatments) that may be of interest.

**Author response 57: We have made this clearer. Yes, our results indeed point that there might be a threshold there.**

**Discussion:**

Reviewer comment 58:

The discussion addresses most of the components of the paper, but in my view, does not thoroughly analyze many of the important findings. In particular, the authors do not discuss their findings that the composition of pollen collected by bees did not differ between treatments. Further, I feel that some unsubstantiated claims are made that should be removed. Namely, the authors state that they have evidence that warming can lead to shifts in the composition of pollen collected by pollinators, which, by my interpretation, is in contradiction with their data.

**Author response 58: Thank you for your comment. We have now fundamentally restructured our discussion, which now we hope addresses your concern (see also Author response 1).**

Reviewer comment 59:

L160: The term ‘Consequently’ does not seem to fit here

**Author response 59: We agree – we have replaced it with nonetheless.**

Reviewer comment 60:

L162-164: This reasoning does not make sense to me, and seems unsubstantiated by the data. This study showed that there was no significant difference in the composition of pollen collected by treatment, and, further, both bees and flowers became active earlier under warming. Thus, I don’t understand why the authors claim that a shift in flowering will change the pollen available to bees under warming. I may be misunderstanding but this should be clarified.

**Author response 60: see also Author response 1. We have also toned down our statement here.**

Reviewer comment 61:
L170-171: Suggest rephrasing to ‘Environmental factors that are known to affect bee foraging – such as temperature and solar radiation – vary...’ Then, it would improve flow to connect this sentence with the rest of the paragraph by, for example, emphasizing the role of temperature in shaping diel rhythms in bee behavior.

**Author response 61: Thank you for this excellent suggestion. We have made the subsequent change.**

Reviewer comment 62:
L173: Suggest omitting ‘clearly’

**Author response 62: We have removed it.**

Reviewer comment 63:
L177: Please clarify the meaning of ‘the effect of time’- on what, and at what scale?

**Author response 63: This sentence was confusing and did not engage nicely with this section of discussion. Thus, we have removed this sentence.**

Reviewer comment 64:
L178-179: I suggest explaining how temperature may not affect bee activity. Otherwise this statement seems to contradict many of the findings in this study.

**Author response 64: This sentence no longer exists.**

Reviewer comment 65:
L179-181: I suggest rephrasing to ‘A more comprehensive understanding of the relative importance of within-hive and ambient temperature to bee foraging behavior could be developed by...’ then describe what kind of study could be done to this end.

**Author response 65: We have rephrased this part of the sentence as the reviewer suggested.**

Reviewer comment 66:
L182-183: I feel that this sentence would fit better earlier in the paragraph.

**Author response 66: We agree – we have removed this sentence and have indicated that our finding is in accordance with previous work earlier in this paragraph.**

Reviewer comment 67:

L183-184: I suggest replacing ‘early in the morning’ with ‘at 6:00’ and remove ‘at 6:00’ from later in the sentence

**Author response 67: We have made the suggested change.**

Reviewer comment 68:

L185: If true, I suggest replacing ‘not evident’ with ‘not captured by the range of temperatures in this study’

**Author response 68: We agree with the reviewer that this modification explains it better.**

Reviewer comment 69-71:
L185-186: I suggest omitting ‘Turning to foraging efficiency per individual’, the replace ‘the efficiency’ with ‘foraging efficiency’
L190: I suggest adding ‘early-foraging’ after ‘since
L191: I suggest add ‘pollen’ before ‘quantities’

**Author response 69-71: We are thankful for these comments, which we have made suggested changes.**

Reviewer comment 72:
L194-197: I suggest rephrasing this sentence to improve clarity, perhaps by moving the information on the generality of honey bees to a separate sentence. L197-198: Because this was not tested in this study, it may be helpful to include a reference here.

**Author response 72: Thank you for the suggestion. We have separated the information about honey bees being generalist pollinators into a standalone sentence. We have also now provided a reference here to support this statement.**

Reviewer comment 73:
L199: For clarity, please add ‘floral’ before ‘resources’ and ‘landscape’

**Author response 73: We have now added ‘floral’ here.**

Reviewer comment 74:

L200-202: I am unclear on how the authors have come to this conclusion based on the results of this study.

**Author response 74: We agree. We have now removed this conclusion.**

Reviewer comment 75:

L208-210: I suggest citing these concepts.

**Author response 75: We have now provided references in association with these concepts.**

Reviewer comment 76:

L216-219: I suggest connecting this sentence to the remainder of the paragraph

**Author response 76: We have now made changes to this paragraph to strengthen the logical flow.**

Reviewer comment 77:

L216: Please change ‘nutritional values’ to ‘nutrients’

**Author response 77: We have changed it to nutrients.**

Reviewer comment 78:

L220-222: I suggest rephrasing to ‘development of honey bees [if true] due to the lack of necessary nutrients (Filipiak et al., 2017), although access to a single flowering species with protein-rich pollen may be more important than high floral diversity for larval growth...’ L227-

**Author response 78: We have rephrased this sentence.**

Reviewer comment 79:

228: This seems important, and it may be helpful to address this concept sooner. L229-231: Please rephrase to emphasize that this study’s findings are in contrast with those of Tasei and Aupinel.

**Author response 79: We have now rephrased these sentences to emphasize differences in our findings.**

Reviewer comment 80:

L233: I suggest clarifying what is meant by ‘changes in’

**Author response 80: We have now made it clear as the composition and abundance.**

Reviewer comment 81:

L234: I suggest replacing ‘are determined by several factors, such as’ with ‘change across’

**Author response 81: We have made the suggested change.**

Reviewer comment 82:

L235-236: I suggest rephrasing to ‘Since __ months passed between our field and larvae feeding experiments...’

**Author response 82: We have made the suggested change.**

Reviewer comment 83:

L237: I suggest omitting ‘in’, add ‘the’ before ‘larval’, and change ‘experiments’ to ‘experiment’ L238: I suggest adding ‘that were more common’ after ‘that’

**Author response 83: We have made these suggested changes.**

Reviewer comment 84:

L239: I suggest omitting this line as it is implied

**Author response 84: This line has now been removed.**

Reviewer comment 85:

L241: Please clarify the meaning of ‘seasonally consistent’

**Author response 85: Thank you for your comment. By “seasonally consistent,” we mean that *B. pilosa* is present and blooms throughout different seasons, providing a continuous pollen source across the year. This stable presence allows bees to rely on *B. pilosa* as a regular food source, which may help in maintaining a consistent microbial community within their gut, as it provides an unchanging nutritional input over time. We have now rephrased this sentence.**

Reviewer comment 86:

L241-242: Please describe how this may occur

**Author response 86: See also Author response 85.**

Reviewer comment 87:

L243-244: Please describe what is meant by ‘larvae grew heavier’

**Author response 87: Thank you for pointing this out. By “larvae grew heavier,” we mean that the larval bees in our study attained a higher body mass when fed a diet with an increased proportion of *B. pilosa* pollen. We have now elaborated this point.**

Reviewer comment 88:

L244: Expand on how a pollen-only diet could affect larval resilience

**Author response 88: We appreciate this suggestion. A pollen-only diet may impact larval resilience by potentially limiting the diversity of nutrients and secondary metabolites that larvae would otherwise obtain from a mixed diet. This limited nutrient diversity might weaken their immune responses, making them more susceptible to stressors like viruses, pesticides, or parasites. We have expanded this point in the revised text.**

Reviewer comment 89:

L245: Please describe what ‘other developmental factors’ should be evaluated

**Author response 89: Thank you for this comment. By “other developmental factors,” we refer to elements such as larval immune responses and gut microbiota diversity. We have now elaborated this point.**

Reviewer comment 90:

L246: There seems to be text missing after ‘consequences’

**Author response 90: Thank you for noticing this. The intended text was, ‘under varying dietary compositions’.**

Reviewer comment 91:

L249: This section does a good job in addressing the small sample size of this study, but does not touch on the issue of any lingering effects in hives the day after they were used in the opposite treatment. To me, this seems like an important angle.

**Author response 91: Thank you for your concern. This is possible, and we have incorporated this possibility in the discussion.**

Reviewer comment 92:

L254: To me, ‘physiological changes’ include an increase in metabolism driven by high temperatures. Perhaps the authors intended to discuss acclimatization to warming.

**Author response 92: Indeed, we clarified in our revised texts that we focus on potential acclimatization rather than short-term metabolic responses.**

Reviewer comment 93:

L258: I suggest rephrasing to ‘hives to test the robustness of our findings’

**Author response 93: Thank you – we have made the suggested change.**

Reviewer comment 94:

L261: I suggest clarifying what has occurred over the past decade that has made studying these trends more important

**Author response 94: We have rephrased this paragraph fundamentally to explain that studying climate change impact on plant-pollinator interactions has gained importance over the past decade.**

Reviewer comment 95:

L265-267: From my reading of this manuscript, I disagree that the data, as currently presented, supports the idea that warming changes the composition of pollen collected by honey bees.

**Author response 95: Thank you for your comment, and as those in Author response 1, 56, and 60. We have toned down the language used.**

**Methods:**

The methods are mostly clear. However, I have several line edits and concerns about pseudoreplication and model selection, which I describe below.

Reviewer comment 96:

L273: Though this was addressed in the discussion, I am still concerned that there is a high level of pseudoreplication in this study given that only two hives were used

**Author response 96: Thank you very much for your comments on the potential issue of pseudoreplication, which we certainly are aware of, as we have included in the discussion.**

**In our study, the limitation of using only two beehives may limit the generalizability of the results. While we recognize the limitation due to the small number of hives, this study was intended as a preliminary investigation. Our findings offer initial insights into the effects of warming on beehive activity, which can guide future studies with larger sample sizes.**

**To minimize the potential influence of pseudoreplication, our detailed and repeated observations over the study period may account for temporal variability and behavioral patterns, allowing for a more nuanced understanding of hive responses to warming. In our analysis, we also considered beehive id as a random effect to account for the inherent variability within each hive.**

**We present this study as a pilot to provide insights into future. We hope this will guide further experimental work involving more replicates to confirm and extend our findings.**

Reviewer comment 97:
L275: Please state which pollen substitutes were used

**Author response 97: The pollen substitutes are typically used by beekeepers. In our case, we combined a wide variety of bee pollen, soybean powder, and water, and mix them thoroughly as soft dough.**

Reviewer comment 98:
L277: Please describe why hives were heated for 20 hours and not 24

**Author response 98: Thank you for pointing this out. The decision to heat the beehive for 20 hours, rather than 24, was due to a logistical constraint. Specifically, the heating mat used to warm the hive was powered by a portable charger with a battery life limited to 20 hours. Unfortunately, this limitation prevented us from maintaining continuous heating for the full 24-hour period.**

Reviewer comment 99:

L277: ‘beehive’ should be plural here, I believe

**Author response 99: We suppose this comment was for L122, which we have now made it plural.**

Reviewer comment 100:
L292: Why one to two hours, and not every hour? Please clarify.

**Author response 100: Thank you for the comment. Based on our preliminary results, we observed that foraging activities have decreased significantly in the afternoon. Since our primary focus was on the peak activity period, i.e. 6:00-10:00, we opted to sample foraging behavior once every two hours from 10:00-16:00 when activity levels were lower.**

Reviewer comment 101:

L293: Please add the number of total days of the experiment here for clarity

**Author response 101: It’s a total of 26 days. We have now added this information.**

Reviewer comment 102:

L294-296: Please describe how observers knew which bees were starting orientation flights

**Author response 102: During the 5-minute observation periods, the observer carefully monitored bee behaivor. Whenever bees were found to conduct orientation flights, that specific 5-minute observation was excluded from the analysis. Thus, there were any issue of misidentifying bee activity as the observations during such flights were not included in the data. We have now rephrased this sentence to make it clearer (L138-141).**

Reviewer comment 103:

L298: I suggest omitting the ‘the’ before ‘ImageJ’

**Author response 103: We have now removed ‘the’.**

Reviewer comment 104:

L299: To cite ImageJ, I believe one should use Schneider et al. 2012 (https://doi.org/10.1038/nmeth.2089). Also, I suggest starting a new paragraph at the end of this sentence.

**Author response 104: We have cited Schneider et al. 2012, and have also started as a new paragraph starting from ‘In addition’.**

Reviewer comment 105:

L300-301: I suggest describing what pollen traps are and how much pollen they allow one to observe of what the bees have collected

**Author response 105: The pollen traps are widely and commercially used by beekeepers. Pollen traps are placed at the entrance of the beehive. When the bees pass through the black hole of the trap, the pollen on their legs is scraped off. The pollen then falls into the box below. We have now made it clearer.**

Reviewer comment 106:

L302: Please explain why pollen was collected every one to two hours at these different intervals

**Author response 106: See also Reviewer comment 100. We collected the pollen in accordance with our sampling of bee foraging behavior.**

Reviewer comment 107:
L304: Please clarify the meaning of ‘standardized per hour’

**Author response 107: Some pollen loads were sampled once per hour, while others were sampled every two hours. To ensure consistency, we standardized the pollen loads across all observation periods to represent an hourly rate. This means that for periods with two-hour sampling, the total pollen load was divided by two, effectively standardizing the data per hour for comparison across all sampling intervals. We have rephrased this sentence to make it clearer.**

Reviewer comment 108:

L305: Please describe what an observation entails in this context

**Author response 108: We agree that this is unclear. We have rephrased it.**

Reviewer comment 109:

L307: I suggest replacing ‘refrigerator’ with ‘freezer’ and clarifying what further investigation was performed

**Author response 109: We have replaced refrigerator with freezer. We have removed ‘further investigation’, by which we meant experiment 3.**

Reviewer comment 110:

L317: ‘pollen’- does this refer to a pollen grain or the entirety of that species’ pollen? Please clarify.

**Author response 110: You are absolutely right that the pollen here actually means pollen grain. We have added it now.**

Reviewer comment 111:

L320: Reference not found

**Author response 111: By this we referred to Table S1. Due to the formatting issue, it should be fixed now.**

Reviewer comment 112:

L340: I suggest omitting ‘the’

**Author response 112: We have removed ‘the’.**

Reviewer comment 113:

L344: Please include the family that *B. pilosa* belongs to

**Author response 113: It belongs to Asteraceae, which we have added now.**

Reviewer comment 114:

L346: Reference not found

**Author response 114: It’s Table S1.**

Reviewer comment 115:

L349: Please describe why these growing conditions were chosen

**Author response 115: This condition was chosen since it reflects a typical ambient condition in Taiwan, which can trigger seed germination of *B. pilosa.***

Reviewer comment 116:

L351: Please clarify whether plants were moved on the 17th day of seedling growth or 17 days after planting

**Author response 116: We meant ‘on the 17^th^ day of seedling growth. We have now clarified it.**

Reviewer comment 117:

L355-356: Please describe how these temperatures were chosen

**Author response 117: The temperature of control group was selected to match the average conditions observed in Experiment 1. The warming group was set to 30/25**℃**, as this was the nearest available warming condition within the phytotron facilities at National Taiwan University.**

Reviewer comment 118:
L357: Suggest changing ‘would be’ to ‘were’

**Author response 118: We have changed it to ‘were’.**

Reviewer comment 119:
L358-360: Please state how much fertilizer was used

**Author response 119: ~300 mL of fertilizer was used, with Huabao No. 2 and No. 3 diluted by 1000 times prior to use.**

Reviewer comment 120:
L360: Please clarify how many individuals had begun flowering when the observations began

**Author response 120: This information has already been provided in L211.**

Reviewer comment 121:

L362: I suggest rephrasing to ‘that had just started their first bloom or finished their last bloom’

**Author response 121: We have made the suggested changes.**

Reviewer comment 122:

L363: Why was there a difference in the number of individuals in control versus warming?

**Author response 122: This was because some plant individuals had just started their first bloom and were thus not used, resulting in difference in the number.**

Reviewer comment 123:

L366: ‘anther’ should be plural here

**Author response 123: This term was not used anymore.**

Reviewer comment 124:

L375: Please clarify what ratio the semi-artificial diet was in

**Author response 124: This information has already been provided in L252-259.**

Reviewer comment 125:

L378: Reference not found

**Author response 125: This was Table S2.**

Reviewer comment 126:

L381: Please clarify how this equation was developed, and why it is of use here

**Author response 126: We believe that the explanation provided in L231-236 adequately describes the development of this equation. The purpose of this equation is to adjust the relative proportions of various plant pollen types, allowing us to create distinct differences in pollen composition across the four pollen composition treatments.**

Reviewer comment 127:

L395: Please clarify what further experiments that are referenced. If they are not otherwise mentioned in this study then I would suggest removing this.

**Author response 127: By this we meant Experiment 3.**

Reviewer comment 128:

L397: Here and elsewhere I believe this should be formatted as ‘from Hendriksma et al. (2011).’

**Author response 128: Thank you for spotting this. We have made subsequent changes.**

Reviewer comment 129:

L406: ‘could’ to ‘was used to’ if this is the case

**Author response 129: We have changed it to ‘was used to’.**

Reviewer comment 130:

L407: Please describe why there was a change in feeding regimen after day 1

**Author response 130: This change was based on the protocol developed by Hendriksma et al. (2011).**

Reviewer comment 131:

L411: Extra space after ‘-20’, and ‘refrigerator’ should be ‘freezer’

**Author response 131: We have changed it to ‘freezer’.**

Reviewer comment 132:

L413: ‘According’ is not the appropriate term here from my understanding

**Author response 132: We have now changed it by using ‘Following the methodology of’ for clarity.**

Reviewer comment 133:

L413: Please clarify what diet was given

**Author response 133: The details of the semi-artificial diet were provided in L252-259.**

Reviewer comment 134:

L414: Please describe why the volume of food was increased over time

**Author response 134: This also follows the protocol developed by Hendriksma et al. (2011).**

Reviewer comment 135:

L416: Please describe the intervals at which the larvae were fed

**Author response 135: We have now made it clear in L265-267.**

Reviewer comment 136:

L417: Omit ‘meticulously’

**Author response 136: We have removed it.**

Reviewer comment 137:

L419: I do not think that parentheses are needed here

**Author response 137: They have been removed.**

Reviewer comment 138:

L425: This citation is out of date, I believe

**Author response 138: We have now provided an updated citation associated with it.**

Reviewer comment 139:

L429: Why was a VIF of <5 chosen? This seems high. Please cite.

**Author response 139: Thank you for your concern. While some studies use stricter thresholds (e.g., 2.5 or 3), a VIF value below 5 is also widely accepted as a threshold where multicollinearity is unlikely to significantly affect the model’s estimates. This threshold is supported by various sources, such as Akinwande et al. (2015). Hence, we consider it an appropriate choice for assessing multicollinearity in our study.**

Reviewer comment 140:

L424-430: Was any model selection performed? Given the large number of predictors, I feel this may be an important step in analyzing these data in a non-biased fashion.

**Author response 140: Thank you – indeed we have conducted model selection based on AIC values. We have now provided these details in L275-278.**

Reviewer comment 141:

L439-441: This sentence brings up many questions- why was the third degree included, and how did the authors make the decision to reduce this degree? Again, was model selection being used?

**Author response 141: See also Author response 140. We have now provided this information.**

Reviewer comment 142:

L442: Were daily averages of ambient temperature and colony size used? Please clarify.

**Author response 142: Yes, they are included in the model. We have rephrased the sentence for clarity.**

Reviewer comment 143:

L443: This analysis needs to be fully described.

**Author response 143: We have rephrased it and made it much clearer now.**

Reviewer comment 144:

L448: Suggest rephrasing to ‘To compare the composition of pollen collected by bees under control...’

**Author response 144: We have changed this sentence as suggested.**

Reviewer comment 145:

L455-461: The authors may also consider testing whether anthesis alone was significantly earlier in warming treatments by simply testing for a difference in average anthesis time between treatments.

**Author response 145: This might be interesting to explore. However, we believe that our current analysis, which tracks temporal changes in the number of florets at anthesis, provides a more comprehensive understanding of how warming affects the phenology of the plants over time. Simply testing for a difference in average anthesis timing between treatments would miss the nuance of these temporal dynamics and the detailed changes occurring throughout the day.**

Reviewer comment 146:

L458-459: Please further describe why the second degree was used. L459: I suggest omitting ‘different’

**Author response 146: See also Author response 140. Here we have compared model fit using AIC values. We have also now removed ‘Different’.**

Reviewer comment 147:
L464: I suggest omitting ‘the’ before ‘larval’

**Author response 147: We have now removed ‘the’.**

Reviewer comment 148:

L466: I suggest omitting ‘Different’

**Author response 148: We have now removed ‘Different’.**

**Figures and tables:**

Reviewer comment 149:

Across all figures and tables, please ensure that species names are italicized.

**Author response 149: We have now checked all figures and tables and ensured that species names are now all italicized.**

Reviewer comment 150:

Fig. 1: Standard error shading would help with the interpretation of these curves, as would a more thorough description of how the curves were produced.

**Author response 150: We have now added the confidence intervals; as we have already pointed out, the lines are predicted relationships from generalized linear mixed models.**

Reviewer comment 151:

Fig. 2: Missing a legend for colors. Please also describe how these curves were fit and add standard error shading.

**Author response 151: We have now added a legend associated with this figure. We have also now provided the confidence intervals.**

Reviewer comment 152:

Fig. 3: Please describe how these curves were fit and add standard error shading. Also, the phrase ‘total number of anthesis onset’ is unclear, please rephrase. Finally, it may be better to include the actual time of peak flowering anthesis.

**Author response 152: Similarly, we have provided the confidence intervals. As reviewer comment 29 and 52 suggested, ‘Number of anthesis onset’ is no longer used. We have now phrased it as ‘number of florets at anthesis’, which is more accurately in accordance with the biology of *B. pilosa.***

Reviewer comment 153:

Fig. 4: For (A), box plots would be more appropriate in my opinion. In the caption, it would be more clear to place the letters (A) and (B) before their respective descriptions. Also, please include a legend describing the colors in (B). Finally, please minimize the overlap among colors between (A) and (B).

**Author response 153: Thank you for your comments. We have now illustrated (A) using box plots. We have also placed (A) and (B) before their respective descriptions. We have now provided (B) a legend. In terms of use of colors, we have reduced their overlap to avoid confusion.**

**In addition, to maintain the order of (A) and (B) being referred to in the paper, we have swapped their order: previous (A) now becomes (B), whereas previous (B) now becomes (A).**

Reviewer #2: The manuscript has significant value in bee management. However, vigorous revision is needed. A table containing a list of bee-foraged plants (with relative frequencies) will enrich the manuscript. My specific comments are given below:

Reviewer comment 154:

Line 50: Pollinators and plants must match phenologically----- mean? Rewrite clearly. Diverse insect species pollinate most flowering plants. However, all are not equally effective. The pollination efficiency of a pollinator depends on its foraging strategy and floral characteristics (Layek et al. 2022 Journal of Asia-Pacific Entomology 25: 101882. Doi: 10.1016/j.aspen.2022.101882).

**Author response 154: We appreciate this insightful comment. Yet we have significantly restructured this paragraph, following the reviewer 1 comments. Hence, this comment is no longer relevant.**

Reviewer comment 155:
Line 51-52: Bees (Hymenoptera: Anthophila) are one of the most ecologically and economically important pollinators worldwide (Hristov et al., 2020)----- add more references.

**Author response 155: We have now replaced it with three more key references in association with this statement.**

Reviewer comment 156:
Line 158-159: results showed that warming does change bee foraging behavior within the day, with more foragers departing from beehives and carrying back more pollen in the early morning. ----- Much literature is available regarding this issue (e.g., Forcone et al. 2011 Grana 50: 30-39. Doi: 10.1080/00173134.2011.552191; Tan et al. 2012 Apidologie 43: 618-623. doi 10.1007/s13592-012-0136-y; Layek et al. 2020 Palynology 44: 114-126. Doi: 10.1080/01916122.2018.1533898). Consider these.

**Author response 156: We have now cited two relevant references in line with this statement.**

Reviewer comment 157:
Line 172-173: pollen weight increasing with temperature----- Weight of individual corbicular pollen load? Or total pollen collection per unit of time? It needs to be clearly described throughout the manuscript. The amount of pollen collection greatly depends on the hive's surrounding pollen availability and the colony’s demand.

**Author response 157: Thank you for your comment. By this we meant the weight of pollen loads that were collected by all foragers at a given time, rather than individual. We have made this clearer and elsewhere in the manuscript.**

Reviewer comment 158:

Line 160: foraging efficiency per individual of bees remained unaffected by warming. ----- Did they measure the foraging efficiency of an individual worker? If yes, it needs to be detailed in the Material & method and Result section.

**Author response 158: No, we did not measure the foraging efficiency for individual workers, since this is logistically very difficult to measure. We measured them as a group. This information has already been provided in L152-154. In addition, we have now termed foraging efficiency as pollen collection rate.**

Reviewer comment 159:

Line 293: eight 5-minute observations each day of the experiment------ eight observations taken throughout the daytime at regular intervals?

**Author response 159: We believe that we were clear about this methodology in Experiment 1.**

Reviewer comment 160:

Line 304-306: For each observation, we also determined the foraging efficiency per individual by dividing pollen weight by the number of foragers recorded from each 5-minute footage.---- It is just the number of returning pollen foragers. All foragers do not collect pollen at a given time. So, it is not actual foraging efficiency. Therefore, avoid the term ‘foraging efficiency’.

**Author response 160: Thank you for this insightful comment. We have now replaced the term foraging efficiency with pollen collection rate. Besides, we rephrased these sentences in L152-154 to make it clearer (see also Author response 158).**

Reviewer comment 161:
Line 423-469: statistical analysis: Rewrite more compactly.

**Author response 161: Thank you – we have now made significant changes, which are now more comprehensive (as we have addressed several of reviewer 1 comments related to statistical analysis).**
